# Pediatric hydrocephalus outcomes: a review

**DOI:** 10.1186/2045-8118-9-18

**Published:** 2012-08-27

**Authors:** Matthieu Vinchon, Harold Rekate, Abhaya V Kulkarni

**Affiliations:** 1 Department of Pediatric Neurosurgery, Lille University Hospital, Lille, France; 2 Chiari institute, New York, NY, USA; 3 Division of Neurosurgery, Hospital for Sick Children, Toronto, ON, Canada; 4 Department of Pediatric Neurosurgery, Hôpital Roger Salengro, CHRU de Lille, Lille Cedex 59037, France

**Keywords:** Pediatric hydrocephalus, Outcome, Shunt obstruction, Shunt infection, Mortality, Child-to-adult transition

## Abstract

The outcome of pediatric hydrocephalus, including surgical complications, neurological sequelae and academic achievement, has been the matter of many studies. However, much uncertainty remains, regarding the very long-term and social outcome, and the determinants of complications and clinical outcome. In this paper, we review the different facets of outcome, including surgical outcome (shunt failure, infection and independence, and complications of endoscopy), clinical outcome (neurological, sensory, cognitive sequels, epilepsy), schooling and social integration. We then provide a brief review of the English-language literature and highlighting selected studies that provide information on the outcome and sequelae of pediatric hydrocephalus, and the impact of predictive variables on outcome. Mortality caused by hydrocephalus and its treatments is between 0 and 3%, depending on the duration of follow-up. Shunt event-free survival (EFS) is about 70% at one year and 40% at ten years. The EFS after endoscopic third ventriculostomy (ETV) appears better but likely benefits from selection bias and long-term figures are not available. Shunt infection affects between 5 and 8% of surgeries, and 15 to 30% of patients according to the duration of follow-up. Shunt independence can be achieved in 3 to 9% of patients, but the definition of this varies. Broad variations in the prevalence of cognitive sequelae, affecting 12 to 50% of children, and difficulties at school, affecting between 20 and 60%, attest of disparities among studies in their clinical evaluation. Epilepsy, affecting 6 to 30% of patients, has a serious impact on outcome. In adulthood, social integration is poor in a substantial number of patients but data are sparse. Few controlled prospective studies exist regarding hydrocephalus outcomes; in their absence, largely retrospective studies must be used to evaluate the long-term consequences of hydrocephalus and its treatments. This review aims to help to establish the current state of knowledge and to identify conflicting data and unanswered questions, in order to direct future studies.

## Review

### Introduction and definition of terms

Pediatric hydrocephalus (HC) is a surgical disease. If left untreated, most cases are lethal [1]. With present-day standard of care, most patients with HC will survive; however, death from hydrocephalus still exists [2,3], and the sequelae among long-term survivors are frequent and often severe [4]. Because of the multiplicity of causes of hydrocephalus, associated diseases, complications of treatment, and the inherent complexity of the patient population, reliable data on outcomes are difficult to obtain. The outcome of hydrocephalic patients has been the subject of many studies, some presenting conflicting results, and many focusing on a limited view of this vast field. Among the questions asked by legitimately anxious parents, and sometimes by the patient himself, are: what are the risks of complication of surgery? What are the risks that my child dies of this disease? What are the sequels that my child might suffer? We shall therefore categorize hydrocephalus outcomes under the following broad headings: surgical outcome (shunt half-life, rate of shunt malfunction, rate of infection, and the issue of shunt-independence) and complications of endoscopy; mortality (shunt-related as well as related to other causes); morbidity (cognition, motor system, vision, epilepsy, neuroendocrine problems and fertility, chronic headache); and functional social outcome (schooling, social integration, marital status). The present review aims to discuss these different facets of outcome in pediatric hydrocephalus, review the available literature on the topic and identify the gaps in our present knowledge. We will try to identify predictive factors for outcome and to define what are the medical needs for this population in the future, especially when they become adults, how to evaluate them, and which guidelines can be proposed for follow-up.

### Problems and controversies in outcome definitions

#### Surgical outcome

Shunt failure can be aseptic (malfunction) or septic (shunt infection). Malfunction includes obstruction, overdrainage, underdrainage, and occult shunt failure (including the need for elective revision for tube lengthening). Its occurrence being duration-dependent, the incidence of malfunction is generally expressed as actuarial survival. Reoperation, although potentially subject to surgeon bias, can be seen as the best criterion to define malfunction, as it is a binary variable and a dated event, allowing survival analysis. However, some cases of malfunction can be dealt with without surgery, e.g., overdrainage that is solved by valve adjustment or one that is simply accepted by the patient as a minor nuisance. On the other hand, occult shunt failure (e.g., a broken shunt in an asymptomatic patient) is sometimes accepted with little scientific evidence, as an indication that the patient has become shunt-independent [5]. A more rigorous approach to shunt independence is advocated below.

Shunt infection has been defined differently depending on the goal of the study: a positive culture is an adequate criterion for bacteriological studies; clinical symptoms with positive culture and or pleocytosis (Odio’s criteria) [6] is a criterion more adapted to clinical studies, however cultures can be falsely positive as well as falsely negative. Restrictive definitions excluding cases with negative cultures carry the risk of underestimating the real problem. Studies on surgical outcome should adopt a surgery-based criterion: a medical diagnosis based on clinical findings and/or biology, leading to reoperation or death [7-9]. This definition, based on the assumption that infection cannot be cured without reoperation, generally shunt removal, has the advantage of a binary and dated event allowing actuarial analysis [8]. More detail on the type of infection can be provided using the classification proposed by the CDC [10], rating the infection as incisional (skin and dermis), deep-incisional (subcutaneous tissues), and organ/space infection. The definition of infection by the CDC also emphasizes the importance of clinical judgment: “Diagnosis of an organ/space (surgical site infection) by a surgeon or attending physician” [10]. Although postoperative infection is generally defined as occurring with one month of surgery, the rate of infection should also be evaluated over a longer term, since infection can occasionally occur several years after surgery. Survival analysis should be used in order to take into account the impact of late shunt contamination [9]. Different types of infection rates can be calculated and should be clearly specified: by operation (number of operations complicated by infection); by patient (number of patients in a series having had at least one septic episode); by surgeon (number of septic complications among the total number of patients operated by a given surgeon) [11], or by hospital [12]; and actuarial incidence [8,9]. Rates by surgeon or by hospital can be difficult to ascertain because patients are often operated by different surgeons or in different hospitals [13].

Shunt independence is defined as the successful removal or ligature of all shunts, but it does not always mean that the hydrocephalus is “cured”. It includes patients with successful secondary endoscopic treatment [14,15], but who, nevertheless, remain reliant on internal CSF diversion; patients who become shunt independent spontaneously [16]; and patients who undergo progressive upgrading of an adjustable valve, or planned removal of their shunt, which should be performed with great caution and patience. Some delay after shunt removal is necessary to assert that the patient is truly shunt-independent; how long the follow-up should be is undetermined, but delayed deterioration has been reported several years after apparently successful shunt removal [17]. It should be emphasized that, whereas symptomatic shunt failure proves shunt-dependence, the reverse is not true. Contrary to some authors who stated that the absence of shunt failure during more than 18 years attests to shunt-independence [18], some patients can present with their first malfunction more than 20 years after shunt insertion: in a series of 456 patients having reached adulthood, 22 patients presented with their first shunt failure after the age of 20, with a fatal outcome in one case [4].

Complications of endoscopy include failure (obstruction), infection and surgical morbidity [19]. Failure of endoscopic third ventriculocisternostomy (ETV) can be defined as primary (failure to achieve an efficient stoma) or secondary (obstruction of a previously patent stoma). In studies comparing endoscopy and shunting, failure of the endoscopic technique has also been defined as the need to implant a shunt, but this definition excludes failed cases that were treated by redo ETV [20,21]. Evaluation of the primary failure rate should be based on a clear statement of the intent to treat. The rate of secondary failure is duration dependant, and actuarial survival studies are necessary [22,23]. In spite of a growing literature, the long-term actuarial survival of ETV is still poorly documented. For the comparison between ETV and shunting, important confounders, like age and origin of hydrocephalus need to be accounted for with sophisticated statistical methods [24], although this still falls short of the gold-standard of randomization.

#### Mortality

It is important to determine whether or not mortality is related to hydrocephalus and its treatment. When death occurs immediately after surgery, or of documented shunt failure, shunt-related mortality is beyond doubt. Shunted patients can also die of associated conditions, e.g., brain tumor or metabolic disease, unrelated intercurrent events, e.g., accidental trauma, or related ailment, e.g., malnutrition or infection in a severely debilitated patient. All of these identified causes of death should be considered as shunt-unrelated. There remain grey areas in which the cause of death can be multiple or be totally unexplained. In a shunted patient, the occasional sudden death can often be retrospectively ascribed to shunt complication [25]. Based on these data, sudden unexpected death in a shunted patient should be considered as shunt-related by default, similar to the SUDEP in epilepsy (Sudden Unexpected Death of the Epileptic Patient).

#### Morbidity

This term covers all health-related limitations to living a normal life. It is evaluated quantitatively by outcome and quality of life scales, and by the presence or absence of different sequela (binary variables). Overall functional outcome can be quantified using established generic scales like the Barthel index (10 variables graded 1 to 10) [26], Karnofsky scale (11 grades), and the Glasgow Outcome Scale (GOS, 5 grades) [4]; historically, a five-tier grading system precursory of the GOS had already been proposed by Laurence in 1962 [1], and Resch also used a scale very similar to the GOS [27]. The GOS following the WFNS (1 = normal, 5 = dead) is relatively crude but can easily be calculated in retrospective studies, is not influenced by age, and allows comparisons with other neurosurgical diseases [28]. We found no study on pediatric hydrocephalus using the Rankin 7-grades scale, although this is widely used in adult studies. The Barthel index is a highly detailed quantitative outcome measure, but we found it used in no study on pediatric hydrocephalus. Quality of life can be evaluated quantitatively with SF36 scale [26], which requires a prospective evaluation of patients. Recently, the Hydrocephalus Outcome Questionnaire (HOQ), a disease-specific quality of life scale, has been proposed for pediatric hydrocephalus by Kulkarni *et al.*[29], translated in several languages, and validated by other teams [30]. The Barthel Index, SF36 and HOQ are highly detailed quantitative scores, which require prospective collection of data and a fairly heavy logistic organization.

In hydrocephalus, the most important specific functional concerns are:

· Impaired mobility and ambulation (e.g., cerebral palsy)

· Impaired cognition (mental delay, behavior) [31-33]

· Sensory deficits (vision, hearing) [34-36]

· Endocrine dysfunction (growth, puberty, weight balance, fertility) [4,37,38]

· Epilepsy [8,39]

· Depression [37]

· Pain (chronic headache) [40].

All these variables are often rated dichotomously as present or absent (binary variables) or measured quantitatively, following specific scales. In particular, cognition can be evaluated by different tests, adapted to age: Wechsler preschool, WISC 3, WISC 4 [31,41], Gesell, Bayley developmental scale for infants [33,34,39], and Brunet-Lezine test developmental test for infants [39]. These tests are useful to determine a neuropsychological profile and compare hydrocephalic patients with others. However, many of these tests cannot be performed in severely delayed patients, introducing an important selection bias [42]. Studies reporting the results of tests are conflicting: classically, Dennis *et al.* found a predominant deficiency of performance *versus* verbal IQ [32], while more recent studies did not confirm their results [31,41]. Moreover, these tests often underestimate deficits in social competence [4]. School difficulties can be the result of a range of different deficits, cognitive being the most prominent, as well as frequent absences from school due to repeated hospital stays. Schooling is thus not a faithful reflection of pure cognitive handicap. Schooling is generally grossly rated as normal or delayed [26] or can be given a little more detail, such as: normal, normal with help, delayed, special school for mentally handicapped, and no schooling at all [4].

How a pediatric patient functions in society is perhaps the ultimate test of outcome. This includes professional/career achievement, achievement of intimate relationships, and community integration. It should be evaluated by comparison with the expected social outcomes for peers. In many cases the social outcome is poorer than would be expected based on cognitive deficits and school performance, illustrating a “hidden handicap” [4]. Employment status can be categorized as full employment (on the competitive labor market or independent mothers at home), unemployed, sheltered/assisted employment, and handicapped (at home or in special institution).

### Information gained through existing publications

#### Methods

In order to survey the current state of the outcome literature in pediatric hydrocephalus, a Pubmed search was conducted using “pediatric hydrocephalus” and “clinical outcome” as key words, yielding 206 citations. After selecting clinical series, searching among related citations, and adding some publications on specific topics, the survey consisted of 122 references. These studies included prospective studies, retrospective studies, and cross-sectional surveys. We also identified long-term series, for which patients were followed until late childhood/adulthood. The following data were extracted: the population studied (age and cause of hydrocephalus), duration of follow-up, the incidence of shunt malfunction and infection, the mortality and morbidity, the functional outcome (schooling, occupation, social integration), and in rare studies, the medical resources available.

#### Results

We found only six prospective studies [29,30,34,43-45] and these had a mean follow-up of 20 months [34], 5 years [43], and 9.9 years [29]. In one study, follow-up was terminated when the study endpoint (shunt failure) was reached [46]. Retrospective studies represent the bulk of medical literature on hydrocephalus outcome. The value of these studies rests in the unselected nature of the patient sample, homogeneity of medical management, and long duration of follow-up. Four studies reported on the long-term outcome of patients beyond 18 years [4,26,37,47]. Several studies were based on the voluntary participation of patients [37,47-49]. The willingness to participate in a study, often through the channel of patient advocacy groups, introduces an important selection bias [42]. In addition, some studies presented the neuropsychological evaluation of selected patients [33,41]; their purpose was to define a profile in the hydrocephalus population, rather than an overall view of the outcome. Finally, other studies were based on data stored in registries [12,50], and give an epidemiological rather than a clinical perspective.

To summarize the data from an abundant but heterogeneous and unformatted literature, we constructed two tables. Table 1 is aimed at establishing the incidence and prevalence of the different elements of outcome detailed above, with figures from the literature, and comments on possible bias of the cited studies.

**Table 1:** Incidence of different complications and prevalence of the different types of morbidity according to literature

**Complications and morbidity**		**Data in literature**	**Comment on biases**
shunt obstruction	number of shunt revisions per patient	2.7 at 24.2 years [4] 4.2 at 20 years [26] 2.7 at 17 years [49]	duration-dependent; rates of revision also reflects the closeness of follow-up
	Event-Free Survival at 1 year	70% [8,51] 62% [46] 80% [4]	depends on the age at insertion and the number of prematures shunted
	Event-Free Survival at 10 year	35% [51], 48% [4]	few data on the very long term
Endoscopy failure rate	Event-Free Survival at 5 years	62% [22] 45% [23]	few data on the very long term
shunt infection	per surgery	0.17% [52] 7.9% [11] 8.4% [46]	depends on definition criteria, selection of patients and duration of follow-up
	per patient (long term)	15.6% [4] 37.5% [37]	depends on duration of follow-up
	per surgeon (range within a team)	5.2-12.2% [9] 7–9.4% [53] 6.1-11.7% [11]	significant differences between surgeons [53]; NS[9,11]
	actuarial incidence	8.5% at 1 year [8] 20.3% at 10 years [9]	very few data on long-term incidence
shunt independence	overall	3.2% [54] 9% [55]	depends on etiology of hydrocephalus and eargeness to remove the shunt
	successful endoscopy when shunt malfunction	77% of attempts [15] 64% [14]	reflects selection of patients
Mortality	overall	1.22 [56] 14.6% [57]	depends whether tumors are included or not
	non-tumoral mortality	8.6% [58] 13.7% [49]	depends on duration of follow-up and intercurrent events in debilitated patients
	shunt-related	0 [48] 2.9% [26] 3.65% [57]	depends on duration of follow-up; impact of staff and patient education
Morbidity	motor handicap	30% [30] 34.2% [56] 60% [36]	series with spina bifida
	low IQ	12.5% [47] 54.2% [48]	selection bias for testing
	normal schooling	38% [43] 30% [36] 79% [26]	depends on tolerance of schooling system
	no schooling	5.5% [4] 9% [30] 9% [36]	depends on tolerance of schooling system
	visual	25% [36] 33% [59] 83% [35]	depends on how thoroughly examined
	pregnancy	14.5% [4] 20.6% [37]	impact of early age of shunting
	epilepsy	2%/year [60] 6.5% [4] 14% [30] 25% [57] 30% [36] 32% [39]	associated with severe initial disease and poor outcome
	depression	43.2% [37]	may be biased by selection (self reported morbidity)
	headache	8% [4] 44% [40]	depends on age
social integration	normal job (competitive labor market)	33.3% [4] 56% [5]	bias linked with protracted follow-up
	sheltered job	23% [4,26]	depends on proactive welfare system
	partner	6% [5] 22% [26] 37% [37]	selection bias: studies based on voluntary participation

Table 2 aims at establishing the relationship between predictive variables (including those related to the patient, surgery, and medical resources) and dependent variable (outcome). Four variables (epilepsy, shunt obstruction, shunt infection and endoscopy) are listed both as predictive and as dependent variables. The combination of these variables with themselves is often meaningless (the predictive and dependent variables are collinear), however shunt failure has in itself a definite impact on the risk of further shunt failure. Table 2 shows that there is a broad consensus on some items, such as the association between young age at surgery and shunt failure [8,37,61,62] and shunt infection [7-9,61]. There is a more limited consensus on other items, such as the association between shunt infection and shunt-related mortality [9,58]. For other associations, we found data from only one study (e.g., the correlation between prematurity and quality of life [29]). Other associations had conflicting results (e.g., between shunt obstruction and epilepsy, which was significant according to Bourgeois *et al.*[39], but not Piatt and Carlson [60]). Finally, for several of these associations, we found no documentation in literature (e.g., between epilepsy and social integration). Although a more extensive literature search may fill some of these gaps, there is obviously a great need for more clinical research.

**Table 2:** Association between factors pertaining to the patient, treatments and medical resources (predictive variables) and surgical and clinical outcomes (dependent variables)

**Predictive variable**	**Dependent variable**	**Shunt failure**	**Shunt infection**	**Hydrocephalus-related mortality**	**Endoscopy failure**	**Neurological deficit**	**Epilepsy**	**Cognitive impairment**	**Difficulties at school**	**QOL**	**Social insertion**
Young age at surgery		higher [8,37,61,62]	higher [7-9,61]	higher [50,63]	higher [21,64]	higher [31]	higher [31] NS [60]	higher [31,44]	higher [44]	lower [29,30,37,44]	lower [37]
Prematurity		higher [62,65-68]	higher [7,66-68]	higher [27] NS [67] lower [44,50]	higher [69]	higher [27,44,68]	higher [39]	higher [27,68]	higher [44] NS [9]	lower [29]	no data found
Post-meningitis hydrocephalus		Higher [56,62,70]	NS [7,9]	higher [71]	higher [20] NS [72]	lower [57]	higher [30,39,56]	higher [4,40,56]	higher [5,9,61]	lower [4,30]	lower [4]
ventricular hemorrhage		higher [8,62,70] NS [61]	higher [73] NS [7,9]	lower [4] higher [63]	higher [20,69]	higher [34,57]	higher [39,56,57]	higher [30,34,44,57]	higher [5,61] NS [9]	lower [30] higher [4]	lower [4]
spina bifida		higher [62,74]	higher [74] NS [7]	higher [3,50,58,74]	higher [18,23]	higher [56,57]	lower [39,57]	lower [36,57]	Lower [49] higher [9]	lower [4,30]	lower [4]
shunt obstruction		risk of recurrent revisions [8,62,70]	risk increases with recurrent revisions [70]	main cause [63]	higher [22,23,75]	higher [44] NS [57]	higher [39] NS [60]	higher [57,76] NS [41,47,49]	Higher [76,77] NS [61]	lower [29]	no data found
shunt infection		(1)	(1)	higher [9,58]	higher [22]	higher [30]	higher [9,39] NS [60]	higher [9,57]	higher [9]	lower [9,78]	no data found
endoscopy		lower [19,24]	lower [19,79]	lower [23] NS [73]	(1)	no data found	lower [39,80]	NS [79]	no data found	better [73] NS [79]	no data found
epilepsy		higher in infants [60]	no data found	no data found	no data found	no data found	(1)	higher [36,39,56,81]	higher [39]	lower [29,30]	no data found
local resources		high volume of surgery: lower [12]	high volume of surgery: lower [12]	high volume: lower [12] geographic distance: higher [63]	no data found	no data found	no data found	no data found	no data found	geographic distance: lower [29]	no data found

Because they are both causes and consequences, shunt obstruction, shunt infection and epilepsy are indicated both as predictive and dependent variables.

(1): these correlations are not documented because meaningless (collinear variables); however, shunt obstruction has a documented impact on the risk of recurrent shunt failure [8,62,70].

The voids in this table indicate that there is space for clinical research on hydrocephalus outcome, especially on the very-long term outcome. QOL: quality of life.

### Analysis of information and identification of information gaps

(How can we evaluate the hydrocephalic patient and who does the evaluation?)

#### Self-evaluation by the patient

It is now recognized that the patients’ perspective on their own outcomes is probably the most relevant. The National Institutes of Health has, in fact, made this a strong program focus under Patient-Reported Outcomes Measurement Information System (https://commonfund.nih.gov/promis/). In pediatric hydrocephalus, these outcomes are often completed by proxy (usually parents), because most young children under 10–12 years are not capable of meaningfully answering questions about their health. Health in this context is recognized to be multidimensional and cuts across broad domains of physical health, social-emotional health, and cognitive health. Specific measures of each of these domains exist, with neuropsychological testing being one of the most commonly used. Taken together, these domains comprise the overall concept of health. Quality of life (QOL) is a specific measure of health, taken from the perspective of how the patients feel they are doing in these domains. It can be argued that the ultimate goal of our management is to optimize QOL, since it is the final common measure of outcome. For example, improvements in shunt technology resulting in fewer admissions for shunt failure should be manifest in improved QOL for the patient, as should physiologically optimizing CSF drainage so as to improve intellectual function. While the immediate effects of these could be measured in terms of reduced days of admission or higher neuropsychological test scores, the ultimate test of their impact is the effect they have in improving QOL. Generic measures of overall health and QOL in children include the PedsQL, the Child Health Questionnaire, and the Health Utilities Index, while the Hydrocephalus Outcome Questionnaire is an example of a disease-specific measure [78].

#### Evaluation by clinicians

The literature review found mostly retrospective studies. Evidence-based medicine would require more prospective studies, however these studies have been difficult to set in place and sometimes disappointing in their results and interpretation [43,46]. There are several acknowledged barriers to randomized trials in pediatric hydrocephalus including: a relatively small number of patients, a large variety of available shunt materials and techniques, heterogeneous and often tradition-based practices, and the important questions to be answered (patient development, academic achievement, social integration) require very long-term follow-up. Despite these barriers, we must continue to strive for randomized trials, where appropriate, and to also take steps to make future retrospective and non-randomized prospective studies more useful. For example, scientific societies and journals should encourage the standardized use of evaluation tools and analytic techniques (e.g., actuarial survival rates, QOL scales).

#### What are the determinants of outcome?

The voids in Table 2 show that there is room for clinical research on the determinants of hydrocephalus outcome, especially very-long term outcome. This table can also be expanded by adding other variables not listed above. Among predictive variables not listed because of the paucity of data available, prenatal hydrocephalus is of particular interest, because its predicted outcome is an important element of decision-making for antenatal treatment and/or interruption. Other outcome variables like vision, endocrine sequelae and fertility, are also of importance for the daily life of these patients, and could be anticipated or prevented with better knowledge of their determinants.

#### How can shunt-related mortality and morbidity be reduced?

Table 1 lists a long litany of sequelae and complications, which plague the patients’ life and sometimes make it shorter. Having identified these problems, the next step is to find ways to fix them. Prevention of morbidity requires early diagnosis and treatment of hydrocephalus, but this is often beyond the control of neurosurgeons. The neurosurgeons’ undisputed role is to avoid complications of treatment. As Oakes stated: “Lowering the infection rate and extending the interval between shunt revisions are appropriate and admirable goals. Helping these patients achieve a more useful life is ideal” [42]. Reducing the incidence of shunt infection requires a number of measures before, during and after surgery [7,10], and applying these measures rigorously has proved effective [52]. Analyzing all possible types of shunt failure, Sainte-Rose *et al.* defined the characteristics of the ideal shunt design [51]. However, prospective studies have failed to assert the superiority of one shunt design over another [43,45,46]. Surgery for shunt failure diagnosed at a pre-symptomatic stage has also been advocated as a way to prevent catastrophic shunt blockage and lower the complication rate of shunt revision [82]. Ultimately, the best way to avoid shunt complications might be to employ endoscopy whenever possible, seizing the opportunity when the patient presents with a blocked shunt [14,15]. How often this opportunity translates into a durable benefit for the patient remains to be evaluated [75]. Monitoring the rate of septic and aseptic complications should be part of each neurosurgeon’s duties. Compulsory national registries, like the one created in the UK could become a standard [83]. It would be preferable, however, that the motivation for this comes from clinicians, rather than be imposed by institutional authority.

Another approach to these problems could be through re-organization of healthcare delivery. In-hospital mortality and complications after shunt operation have been shown to be significantly lower in high-volume hospitals, compared to hospitals with a smaller volume of activity [12]. While this would seem to support centralization of care to larger centers, this collides with another important tenet: fast referral in case of emergency [63]. Long distances can also be an impediment to clinical follow-up and Kulkarni *et al.* found a correlation between geographic distance and a lower QOL score [29]. On the other hand, high volume and clinical experience can foster the creation of ad hoc protocols for emergency management [84]. A non-centralized approach would require adequate training and teaching of non-specialist neurosurgeons and the organization of back-up support networks. A side benefit of the latter solution would be a smoother transition to adult care when the child grows up.

#### What are the ideal modalities of follow-up for shunted patients?

The follow-up of hydrocephalic patients is essential, in order to diagnose and manage long-term complications early and to evaluate the results of treatments. There is no consensus regarding the optimal mode of this follow-up. While most authors agree that it falls to the neurosurgeon [85,86], some authors suggest that these patients could be followed by a general practitioner or a pediatrician [87]. As detailed above, however, the complexity and rarity of the condition in general practice often makes the follow-up of these patients a specialist’s role. Standards have yet to be established regarding the frequency of visits and imaging. Several authors advise a clinic visit every second year [82,88]. Fundoscopy is innocuous but has not proved its usefulness for the diagnosis of shunt failure in asymptomatic patients, and is often falsely negative. The role of brain imaging is much debated. It is important to obtain a baseline cerebral image, to provide adequate comparison when suspicion of shunt malfunction arises [18]. This imaging should be performed at least 6 months after shunt insertion or revision in order to show the effect of shunting [45], because it could take up to 14 months for ventricle size to plateau [89]. It appears reasonable to keep X-ray imaging to a minimum and MRI should be preferred to CT whenever possible [90]. MRI poses a different problem in infants who require sedation, even with specific quick sequences [91]. The practice of routine shunt series is based on the possibility of asymptomatic disruption or migration preceding overt shunt malfunction [82], and its yield has been evaluated at 1.4% [92]. Whether such a rate justifies routine shunt series or not is debatable. Up to now, no alternative has been found to explore the physical integrity of the shunt and avoid X-rays.

#### What are the ideal modalities of follow-up after endoscopic surgery?

When ETV became widespread, it was considered, unlike shunts, as a minimally invasive and definitive cure for hydrocephalus. It is now recognized that complications also occur after ETV, including obstruction, infection, CSF leakage, intraventricular hemorrhage, and damage to the tuber cinereum with diabetes insipidus [21,75]. Cases of secondary occlusion of the ventriculostomy with delayed catastrophic deterioration are documented [93], however, estimations of hydrocephalus-related mortality after ETV are lacking. There is no consensus over the modalities of follow-up after ETV. Systematic clinical follow-up appears no less necessary after ETV than for shunted patients. However, the duration of this follow-up is not settled: “At what point follow up can be discontinued is not known” [93]. The need for systematic MRI is debated, some authors considering it unnecessary [94] while the majority recommends its routine use [19,93]. In addition to clinical follow-up, education of the patient and caregivers is mandatory [93]. Long-term clinical follow-up studies are necessary to document the surgical and clinical outcome after ETV.

#### How cost-efficient is the management of pediatric hydrocephalus?

The treatment of hydrocephalus has measureable economic consequences, as does the outcome of such treatment. Economic costs are born by the patient/family, the hospital, the insurer, and society. It is no longer considered beyond the scope of physicians to consider these economic costs in making treatment decisions. In the face of finite healthcare resources, it is our duty to provide the best care, but to also do so in the most economically efficient manner possible. Health care economics is complicated, however, and treatment that might seem cheaper from the perspective of one party (e.g. the insurer), might be more costly to another (e.g., the patient/family).

#### Organizing the transition from child to adult care

Like many pediatric diseases, hydrocephalus begins during childhood and continues during adulthood. The yearly incidence of neonatal hydrocephalus was estimated as 2000 cases in the US [37]; with a mortality of 18.1% during the last four decades [4], the expected population of hydrocephalic patients reaching the age of 20 years should be over 1638 patients every year for the US. The transition that occurs when young patients grow into adults is a complex process, occurring over years, and is often met with sudden changes in health care environment and personnel for these patients. This transition should be anticipated with progressive education of the patient and his family [95]. No universal recipe exists for the management of this transition; the factors involved include the patient’s specific health issues, their ability to cope with complex problems, their ability to independently navigate the healthcare system, the availability of familial support, and the accessibility of healthcare resources. Once transition has occurred, it remains important to measure outcomes relevant to the adult patient, such as achievement in societally-appropriate adult roles (higher education, employment, family, etc.). Knowledge about these outcomes and evidence to improve them is relatively sparse in our literature

## Conclusions

The challenges of measuring and improving outcome of all types in pediatric hydrocephalus have been outlined in this paper. These challenges can only be met with a cooperative, systematic approach that emphasizes methodologically-strong research with long-term follow-up of a broad range of outcomes, all of which are important to patients with hydrocephalus.

## Competing interests

The authors do not have anything to disclose, including commercial, financial, or other associations, that pose a conflict of interest in connection with the submitted article.

## Authors’ contributions

MV: was responsible for the collection of data, interpretation of the data, and for the drafting and editing of the document. HR and AK were responsible for the drafting and editing of the document. All authors read and approved the final manuscript.

## References

[B1] Laurence KM Coates S The natural history of hydrocephalus. Detailed analysis of 182 unoperated cases Arch Dis Child 1962 37 345 362 10.1136/adc.37.194.345 14462827 PMC2012878

[B2] Acakpo-Satchivi L Shannon CN Tubbs RS Wellons JC 3rd Blount JP Iskandar BJ Oakes WJ Death in shunted hydrocephalic children: a follow-up study Childs Nerv Syst 2008 24 197 201 10.1007/s00381-007-0408-4 17594102

[B3] Iskandar BJ Tubbs S Mapstone TB Grabb PA Bartolucci AA Oakes WJ Death in shunted hydrocephalic children in the 1990s Pediatr Neurosurg 1998 28 173 176 10.1159/000028644 9732242

[B4] Vinchon M Baroncini M Delestret I Adult outcome of pediatric hydrocephalus Childs Nerv Syst 2012 [Epub ahead of print]. 10.1007/s00381-012-1723-y PMC3360844 22349961

[B5] Sgouros S Malluci C Walsh AR Hockley AD Long-term complications of hydrocephalus Pediatr Neurosurg 1995 23 127 132 10.1159/000120949 8751293

[B6] Odio C McCracken GH Jr Nelson JD CSF shunt infections in pediatrics. A seven-year experience Am J Dis Child 1984 138 1103 1108 6507392 10.1001/archpedi.1984.02140500009004

[B7] Kulkarni AV Drake JM Lamberti-Pasculli M Cerebrospinal fluid shunt infection: a prospective study of risk factors J Neurosurg 2001 94 195 201 10.3171/jns.2001.94.2.0195 11213954

[B8] Piatt JH Jr Carlson CV A search for determinants of cerebrospinal fluid shunt survival: retrospective analysis of a 14-year institutional experience Pediatr Neurosurg 1993 19 233 242 10.1159/000120738 8398847

[B9] Vinchon M Dhellemmes P Cerebrospinal fluid shunt infection: risk factors and long-term follow-up Childs Nerv Syst 2006 22 692 697 10.1007/s00381-005-0037-8 16534646

[B10] Mangram AJ Horna IP Silver LC Jarvis WR Hospital Infection Control Practices Advisory Committee Guidelines for prevention of surgical infection Infect Control Hosp Epidemiol 1999 20 247 280 10.1086/501620 10219875

[B11] Renier D Lacombe J Pierre-Kahn A Sainte-Rose C Hirsch JF Factors causing acute shunt infection. Computer analysis of 1174 operations J Neurosurg 1984 61 1072 1078 10.3171/jns.1984.61.6.1072 6502235

[B12] Smith ER Butler WE Barker FG 2nd In-hospital mortality rates after ventriculoperitoneal shunt procedures in the United States, 1998 to 2000: relation to hospital and surgeon volume of care J Neurosurg 2004 100 Suppl 2 90 97 14758935 10.3171/ped.2004.100.2.0090

[B13] Albright AL Pollack IF Adelson PD Solot JJ Outcome data and analysis in pediatric neurosurgery Neurosurgery 1999 45 101 106 10.1097/00006123-199907000-00025 10414572

[B14] Baskin JJ Manwaring KH Rekate HL Ventricular shunt removal: the ultimate treatment of the slit ventricle syndrome J Neurosurg 1998 88 478 484 10.3171/jns.1998.88.3.0478 9488301

[B15] Cinalli G Salazar C Mallucci C Yada JZ Zerah M Sainte-Rose C The role of endoscopic third ventriculostomy in the management of shunt malfunction Neurosurgery 1998 43 1323 1327 9848845 10.1097/00006123-199812000-00030

[B16] Longatti PL Carteri A Active singling out of shunt independence Childs Nerv Syst 1994 10 334 336 10.1007/BF00335173 7954503

[B17] Lorber J Pucholt V When is a shunt no longer necessary? An investigation of 300 patients with hydrocephalus and myelomeningocele: 11–22 year follow up Z Kinderchir 1981 34 327 329 7331542 10.1055/s-2008-1063369

[B18] Talamonti G D’Aliberti G Collice M Myelomeningocele: long-term neurosurgical treatment and follow-up in 202 patients J Neurosurg 2007 107 Suppl 5 368 386 18459900 10.3171/PED-07/11/368

[B19] Di Rocco C Massimi L Tamburrini G Shunts vs endoscopic third ventriculostomy in infants: are there different types and/or rates of complications? A review Childs Nerv Syst 2006 22 1573 1589 10.1007/s00381-006-0194-4 17053941

[B20] O’Brien DF Javadpour M Collins DR Spennato P Mallucci CL Endoscopic third ventriculostomy: an outcome analysis of primary cases and procedures performed after ventriculoperitoneal shunt malfunction J Neurosurg 2005 103 5 Suppl 393 400 16302610 10.3171/ped.2005.103.5.0393

[B21] Ogiwara H Dipatri AJ Jr Alden TD Bowman RM Tomita T Endoscopic third ventriculostomy for obstructive hydrocephalus in children younger than 6 months of age Childs Nerv Syst 2010 26 343 347 10.1007/s00381-009-1019-z 19915853

[B22] Fukuhara T Vorster SJ Luciano MG Risk factors for failure of endoscopic third ventriculostomy for obstructive hydrocephalus Neurosurgery 2000 46 1100 1111 10.1097/00006123-200005000-00015 10807242

[B23] Naftel RP Reed GT Kulkarni AV Wellons JC Evaluating the Children’s Hospital of Alabama endoscopic third ventriculostomy experience using the Endoscopic Third Ventriculostomy Success Score: an external validation study J Neurosurg Pediatr 2011 8 494 501 10.3171/2011.8.PEDS1145 22044376

[B24] Kulkarni AV Drake JM Kestle JR Mallucci CL Sgouros S Constantini S Canadian Pediatric Neurosurgery Study Group Endoscopic third ventriculostomy vs cerebrospinal fluid shunt in the treatment of hydrocephalus in children: a propensity score-adjusted analysis Neurosurgery 2010 67 588 593 10.1227/01.NEU.0000373199.79462.21 20647973

[B25] Byard RW Mechanisms of sudden death and autopsy findings in patients with Arnold-Chiari malformation and ventriculoatrial catheters Am J Forensic Med 1996 17 260 263 10.1097/00000433-199609000-00015 8870879

[B26] Paulsen AH Lundar T Lindegaard KF Twenty-year outcome in young adults with childhood hydrocephalus: assessment of surgical outcome, work participation, and health-related quality of life J Neurosurg Pediatr 2010 6 527 535 10.3171/2010.9.PEDS09548 21121726

[B27] Resch B Gedermann A Maurer U Ritschl E Müller W Neurodevelopmental outcome of hydrocephalus following intra-/periventricular hemorrhage in preterm infants: short- and long-term results Childs Nerv Syst 1996 12 27 33 10.1007/BF00573851 8869171

[B28] Jennett B Snoek J Bond MR Brooks N Disability after severe head injury: observations on the use of the Glasgow outcome scale J Neurol Neurosurg Psychiat 1981 44 285 293 10.1136/jnnp.44.4.285 6453957 PMC490949

[B29] Kulkarni AV Shams I Quality of life in children with hydrocephalus: results from the Hospital for Sick Children, Toronto J Neurosurg 2007 107 Suppl 5 358 364 18459898 10.3171/PED-07/11/358

[B30] Platenkamp M Hanlo PW Fischer K Gooskens RH Outcome in pediatric hydrocephalus: a comparison between previously used outcome measures and the hydrocephalus outcome questionnaire J Neurosurg 2007 107 1 Suppl 26 31 17644917 10.3171/PED-07/07/026

[B31] Dalen K Bruarøy S Wentzel-Larsen T Laegreid LM Intelligence in children with hydrocephalus, aged 4–15 years: a population-based, controlled study Neuropediatrics 2008 39 146 150 10.1055/s-0028-1085463 18991193

[B32] Dennis M Fitz CR Netley CT Sugar J Harwood-Nash DC Hendrick EB Hoffman HJ Humphreys RP The intelligence of hydrocephalic children Arch Neurol 1981 38 607 615 10.1001/archneur.1981.00510100035004 6975094

[B33] Venkataramana NK Mukundan CR Evaluation of functional outcomes in congenital hydrocephalus J Pediatr Neurosci 2011 6 4 12 10.4103/1817-1745.85703 21977080 PMC3173913

[B34] Adams-Chapman I Hansen NI Stoll BJ Higgins R NICHD Research Network Neurodevelopmental outcome of extremely low birth weight infants with posthemorrhagic hydrocephalus requiring shunt insertion Pediatrics 2008 121 e1167 e1177 10.1542/peds.2007-0423 18390958 PMC2803352

[B35] Andersson S Persson EK Aring E Lindquist B Dutton GN Hellström A Vision in children with hydrocephalus Dev Med Child Neurol 2006 48 836 841 10.1017/S0012162206001794 16978464

[B36] Hoppe-Hirsch E Laroussinie F Brunet L Sainte-Rose C Renier D Cinalli G Zerah M Pierre-Kahn A Late outcome of the surgical treatment of hydrocephalus Child’s Nerv Syst 1998 14 97 99 10.1007/s003810050186 9579862

[B37] Gupta N Park J Solomon C Kranz DA Wrensch M Wu YW Long-term outcomes in patients with treated childhood hydrocephalus J Neurosurg 2007 106 5 Suppl 334 339 17566197 10.3171/ped.2007.106.5.334

[B38] Proos LA Dahl M Ahlsten G Tuvemo T Gustafsson J Increased perinatal intracranial pressure and prediction of early puberty in girls with myelomeningocele Arch Dis Child 1996 75 42 45 10.1136/adc.75.1.42 8813869 PMC1511676

[B39] Bourgeois M Sainte-Rose C Cinalli G Maixner W Malucci C Zerah M Pierre-Kahn A Renier D Hoppe-Hirsch E Aicardi J Epilepsy in children with shunted hydrocephalus J Neurosurg 1999 90 274 281 10.3171/jns.1999.90.2.0274 9950498

[B40] Rekate HL Kranz D Headaches in patients with shunts Semin Pediatr Neurol 2009 16 27 30 10.1016/j.spen.2009.01.001 19410154

[B41] Lacy M Pyykkonen BA Hunter SJ Do T Oliveira M Austria E Mottlow D Larson E Frim D Intellectual functioning in children with early shunted posthemorrhagic hydrocephalus Pediatr Neurosurg 2008 44 376 378 10.1159/000149904 18703883

[B42] Oakes WJ Long-tem outcome of hydrocephalus Editorial J Neurosurg 2007 106 Suppl 5 333 10.3171/ped.2007.106.5.333 17566196

[B43] Hanlo PW Cinalli G Vandertop WP Faber JA Bøgeskov L Børgesen SE Boschert J Chumas P Eder H Pople IK Serlo W Vitzthum E Treatment of hydrocephalus determined by the European Orbis Sigma Valve II survey: a multicenter prospective 5-year shunt survival study in children and adults in whom a flow-regulating shunt was used J Neurosurg 2003 99 52 57 10.3171/jns.2003.99.1.0052 12854744

[B44] Persson EK Hagberg G Uvebrant P Disabilities in children with hydrocephalus-a population-based study of children aged between four and twelve years Neuropediatrics 2006 37 330 336 10.1055/s-2007-964868 17357034

[B45] Xenos C Sgouros S Natarajan K Walsh AR Hockley A Influence of shunt type on ventricular volume changes in children with hydrocephalus J Neurosurg 2003 98 277 383 10.3171/jns.2003.98.2.0277 12593611

[B46] Kestle J Drake J Milner R Sainte-Rose C Cinalli G Boop F Piatt J Haines S Schiff S Cochrane D Steinbok P MacNeil N Long-term follow-up data from the Shunt Design Trial Pediatr Neurosurg 2000 33 230 236 10.1159/000055960 11155058

[B47] Lindquist B Persson EK Fernell E Uvebrant P Very long-term follow-up of cognitive function in adults treated in infancy for hydrocephalus Childs Nerv Syst 2011 27 597 601 10.1007/s00381-010-1311-y 20972682

[B48] Kokkonen J Serlo W Saukkonen AL Juolasmaa A Long-term prognosis for children with shunted hydrocephalus Childs Nerv Syst 1994 10 384 387 10.1007/BF00335127 7842425

[B49] Lumenta CB Skotarczak U Long-term follow-up in 233 patients with congenital hydrocephalus Childs Nerv Syst 1995 11 173 175 10.1007/BF00570260 7773979

[B50] Chi JH Fullerton HJ Gupta N Time trends and demographics of deaths from congenital hydrocephalus in children in the United States: National Center for Health Statistics data, 1979 to 1998 J Neurosurg 2005 103 Suppl 2 113 118 16370275 10.3171/ped.2005.103.2.0113

[B51] Sainte-Rose C Hoffmann HJ Hirsch JF Shunt failure Concepts Pediatr Neurosurg 1989 9 7 20

[B52] Choux M Genitori L Lang D Lena G Shunt implantation: reducing the incidence of shunt infection J Neurosurg 1992 77 875 880 10.3171/jns.1992.77.6.0875 1432129

[B53] Cochrane DD Kestle JRW The influence of surgical operative experience on the duration of first ventriculoperitoneal shunt function and infection Pediatr Neurosurg 2003 38 295 301 10.1159/000070413 12759508

[B54] Iannelli A Rea G Di Rocco C CSF shunt removal in children with hydrocephalus Acta Neurochir (Wien) 2005 147 503 507 10.1007/s00701-005-0494-6 15838593

[B55] Hemmer R Böhm B Once a shunt, always a shunt? Dev Med Child Neurol 1976 37 suppl 69 73 10.1111/j.1469-8749.1976.tb04282.x 1071082

[B56] Kao CL Yang TF Wong TT Cheng LY Huang SY Chen HS Kao CL Chan RC The outcome of shunted hydrocephalic children Zhonghua Yi Xue Za Zhi (Taipei) 2001 64 47 53 11310371

[B57] Heinsbergen I Rotteveel J Roeleveld N Grotenhuis A Outcome in shunted hydrocephalic children Eur J Paediatr Neurol 2002 6 99 107 10.1053/ejpn.2001.0555 11995963

[B58] Tuli S Tuli J Drake J Spears J Predictors of death in pediatric patients requiring cerebrospinal fluid shunts J Neurosurg 2004 100 Suppl 5 442 446 15287452 10.3171/ped.2004.100.5.0442

[B59] Persson EK Anderson S Wiklund LM Uvebrant P Hydrocephalus in children born in 1999–2002: epidemiology, outcome and ophthalmological findings Childs Nerv Syst 2007 23 1111 1118 10.1007/s00381-007-0324-7 17429657

[B60] Piatt JH Jr Carlson CV Hydrocephalus and epilepsy: an actuarial analysis Neurosurgery 1996 39 722 728 10.1097/00006123-199610000-00014 8880764

[B61] Casey AT Kimmings EJ Kleinlugtebeld AD Taylor WA Harkness WF Hayward RD The long-term outlook for hydrocephalus in childhood. A ten-year cohort study of 155 patients Pediatr Neurosurg 1997 7 63 70 9520077 10.1159/000121229

[B62] Tuli S Drake J Lawless J Wigg M Lamberti-Pasculli M Risk factors for repeated cerebrospinal shunt failures in pediatric patients with hydrocephalus J Neurosurg 2000 92 31 38 10.3171/jns.2000.92.1.0031 10616079

[B63] Bryant MJ McEniery J Walker DG Campbell R Lister B Sargent P Withers TK Baker J Guazzo E Rossato R Anderson D Tomlinson F Preliminary study of shunt related death in paediatric patients J Clin Neurosci 2004 11 614 615 10.1016/j.jocn.2003.09.016 15261232

[B64] Koch-Wiewrodt D Wagner W Success and failure of endoscopic third ventriculostomy in young infants: are there different age distributions? Childs Nerv Syst 2006 22 1537 1541 10.1007/s00381-006-0191-7 16944172

[B65] Appelgren T Zetterstrand S Elfversson J Nilsson D Long-term outcome after treatment of hydrocephalus in children Pediatr Neurosurg 2010 46 221 226 10.1159/000319365 21051921

[B66] Boynton BR Boynton CA Merritt TA Vaucher YE James HE Bejar RF Ventriculoperitoneal shunts in low birth weight infants with intracranial hemorrhage: neurodevelopmental outcome Neurosurgery 1986 18 141 145 10.1227/00006123-198602000-00004 3960289

[B67] James HE Bejar R Gluck L Coen R Merritt A Mannino F Bromberger P Saunders B Schneider H Ventriculoperitoneal shunts in high risk newborns weighing under 2000 grams: a clinical report Neurosurgery 1984 15 198 202 10.1227/00006123-198408000-00009 6483138

[B68] Vinchon M Lapeyre F Duquennoy C Dhellemmes P Early treatment of posthemorrhagic hydrocephalus in low-birth-weight infants with valveless ventriculoperitoneal shunts Pediatr Neurosurg 2001 35 299 304 10.1159/000050441 11786697

[B69] Buxton N Macarthur D Mallucci C Punt J Vloeberghs M Neuroendoscopy in the premature population Childs Nerv Syst 1998 14 649 652 10.1007/s003810050291 9840365

[B70] Lazareff JA Peacock W Holly L Ver Halen J Wong A Olmstead C Multiple shunt failures: an analysis of relevant factors Childs Nerv Syst 1998 14 271 275 10.1007/s003810050223 9694339

[B71] Schoenbaum SC Gardner P Shillito J Infections of cerebrospinal fluid shunts: epidemiology, clinical manifestations, and therapy J Infect Dis 1975 131 543 552 10.1093/infdis/131.5.543 1127260

[B72] Warf BC Dagi AR Kaaya BN Schiff SJ Five-year survival and outcome of treatment for postinfectious hydrocephalus in Ugandan infants J Neurosurg Pediatr 2011 8 502 508 10.3171/2011.8.PEDS11221 22044377

[B73] Quigley MR Reigel DH Kortyna R Cerebrospinal fluid shunt infections. Report of 41 cases and a critical review of the literature Pediatr Neurosci 1989 15 111 120 10.1159/000120455 2702346

[B74] Vinchon M Dhellemmes P Ozek M, Cinalli G, Maixner WJ Hydrocephalus in Myelomeningocele: Shunts and Problems with Shunts The spina bifida: management and outcome 2008 Milan: Springer 215 224

[B75] Hader WJ Walker RL Myles ST Hamilton M Complications of endoscopic third ventriculostomy in previously shunted patients Neurosurgery 2008 63 1 Suppl 1 ONS168 ONS174 18728596 10.1227/01.neu.0000335032.31144.17

[B76] Hetherington R Dennis M Barnes M Drake J Gentili F Functional outcome in young adults with spina bifida and hydrocephalus Childs Nerv Syst 2006 22 117 124 10.1007/s00381-005-1231-4 16170574

[B77] Hunt GM Oakeshot P Kerry S Link between the CSF shunt and achievement in adults with spina bifida J Neurol Neurosurg Psychiatr 1999 67 591 595 10.1136/jnnp.67.5.591 10519863 PMC1736621

[B78] Kulkarni AV Drake JM Rabin D Dirks PB Humphreys RP Rutka JT Measuring the health status of children with hydrocephalus by using a new outcome measure J Neurosurg 2004 101 Suppl 2 141 146 15835100 10.3171/ped.2004.101.2.0141

[B79] Kulkarni AV Hui S Shams I Donnelly R Quality of life in obstructive hydrocephalus: endoscopic third ventriculostomy compared to cerebrospinal fluid shunt Childs Nerv Syst 2010 26 75 79 10.1007/s00381-009-0983-7 19714338

[B80] Kramer U Kanner AA Siomin V Harel S Constantini S No evidence of epilepsy following endoscopic third ventriculostomy: a short-term follow-up Pediatr Neurosurg 2001 34 121 123 10.1159/000056006 11359099

[B81] Gathura E Poenaru D Bransford R Albright AL Outcomes of ventriculoperitoneal shunt insertion in Sub-Saharan Africa J Neurosurg Pediatr 2010 6 329 335 10.3171/2010.7.PEDS09543 20887104

[B82] Vinchon M Fichten A Delestret I Dhellemmes P Shunt revision for asymptomatic failure: surgical and clinical results Neurosurgery 2003 52 347 353 10.1227/01.NEU.0000043932.84900.70 12535363

[B83] O’Kane MC Richards H Winfield P Pickard JD The United Kingdom Shunt Registry Eur J Pediatr Surg 1997 7 Suppl 1 56 9497131

[B84] Chern JJ Macias CG Jea A Curry DJ Luerssen TG Whitehead WE Effectiveness of a clinical pathway for patients with cerebrospinal fluid shunt malfunction J Neurosurg Pediatr 2010 6 318 324 10.3171/2010.7.PEDS09534 20887102

[B85] Buxton N Punt J Failure to follow patients with hydrocephalus shunts can lead to death Br J Neurosurg 1998 12 399 401 10.1080/02688699844574 10070440

[B86] Hockley AD Walsh AR Sgouros S Follow-up of children with shunted hydrocephalus (letter) Br J Neurosurg 1996 10 525 8922717

[B87] Kimmings E Kleinlugtebeld A Casey AT Hayward RD Does the child with shunted hydrocephalus require long-term neurosurgical follow-up? Br J Neurosurg 1996 10 77 81 10.1080/02688699650040557 8672262

[B88] Colak A Albright AL Pollack IF Follow-up of children with shunted hydrocephalus Pediatr Neurosurg 1997 27 208 210 10.1159/000121253 9577975

[B89] Tuli S O’Hayon B Drake J Clarke M Kestle J Change in Ventricular Size and Effect of Ventricular Catheter Placement in Pediatric Patients with Shunted Hydrocephalus Neurosurgery 1999 45 1329 1335 10.1097/00006123-199912000-00012 10598700

[B90] Steinbok P Boyd M Flodmark CO Cochrane DD Radiographic imaging requirements following ventriculoperitoneal shunt procedures Pediatr Neurosurg 1995 22 141 146 10.1159/000120892 7786807

[B91] Iskandar BJ Sansone JM Medow J Rowley HA The use of quick-brain magnetic resonance imaging in the evaluation of shunt-treated hydrocephalus J Neurosurg 2004 101 Suppl 2 147 151 15835101 10.3171/ped.2004.101.2.0147

[B92] Vassilyadi M Tataryn ZL Alkherayf F Udjus K Ventureyra EC The necessity of shunt series J Neurosurg Pediatr 2010 6 468 473 10.3171/2010.8.PEDS09557 21039171

[B93] Drake J Chumas P Kestle J Pierre-Kahn A Vinchon M Brown J Pollack IF Arai H Late rapid deterioration after endoscopic third ventriculostomy: additional cases and review of the literature J Neurosurg 2006 105 Suppl 2 118 126 16922073 10.3171/ped.2006.105.2.118

[B94] Mohanty A Vasudev MK Sampath S Radhesh S Sastry Kolluri VR Failed endoscopic third ventriculostomy in children: management options Pediatr Neurosurg 2002 37 304 309 10.1159/000066310 12422045

[B95] Vinchon M Dhellemmes P The transition from child to adult in neurosurgery Adv Tech Stand Neurosurg 2007 32 3 24 10.1007/978-3-211-47423-5_1 17907472

